# Stratified distributional analysis—a novel perspective on RT distributions

**DOI:** 10.1177/17470218241288516

**Published:** 2024-12-03

**Authors:** Rüdiger Thul, Joseph Marsh, Ton Dijkstra, Kathy Conklin

**Affiliations:** 1 School of Mathematical Sciences, University of Nottingham, Nottingham, UK; 2 Donders Institute for Brain, Cognition and Behaviour, Radboud University, Nijmegen, The Netherlands; 3 Centre for Research in Applied Linguistics, School of English, University of Nottingham, Nottingham, UK

**Keywords:** Response times, distributional analysis, lexical decisions, Bayesian modelling

## Abstract

Response times and their distributions serve as a powerful lens into cognitive processes. We present a novel statistical methodology called stratified distributional analysis (SDA) to quantitatively assess how key determinants of response times (word frequency and length) shape their distributions. Taking advantage of the availability of millions of lexical decision response times in the English Lexicon Project and the British Lexicon Project, we made important advances into the theoretical issue of linking response times and word frequency by analysing RT distributions as a function of word frequency and word length. We tested these distributions against the lognormal, Wald, and gamma distributions and three measures of word occurrence (word form frequencies obtained from subtitles and contextual diversity as operationalised as discourse contextual diversity and user contextual diversity). We found that the RT distributions were best described by a lognormal distribution across both megastudies when word occurrence was quantified by a contextual diversity measure. The link between the lognormal distribution and its generative process highlights the power of SDA in elucidating mechanisms that govern the generation of RTs through the fitting of probability distributions. Using a hierarchical Bayesian framework, SDA yielded posterior distributions for the distributional parameters at the single-participant level, enabling probabilistic predictions of response times as a function of word frequency and word length, which has the potential to serve as a diagnostic tool to uncover idiosyncratic features of word processing. Crucially, while we applied our parsimonious methodology to lexical decision response times, it is applicable to a variety of tasks such as word-naming and eye-tracking data.

Word processing has been a significant area of research in the cognitive psychology of language and cognitive neuroscience (Balota, Yap, Hutchison, & Cortese, 2012). For decades, response times (RTs) to words have been used extensively as the major dependent variable in various tasks to gauge the cognitive demands of processing words, with longer RTs indicating a higher cognitive load and more time-consuming processing (Luce, 1986; Ratcliff & McKoon, 2008). Among the many factors that determine RTs, word frequency and word length play a prominent role (e.g., Monsell, 1991; Whaley, 1978). In the current research, we present a novel RT analysis method called stratified distributional analysis (SDA), which extracts substantially more information about the impact of word frequency on RTs at different word lengths than traditional approaches. Crucially, while the present analysis focusses on word frequency and word length, SDA is a versatile framework that can provide quantitative answers to theoretically important questions, including how other word properties such as age of acquisition and neighbourhood densities modulate RTs (Dijkstra & Peeters, 2023). Consequently, SDA may bring researchers closer to accurately predicting RTs based on a word’s properties, and in so doing, reveal something about the underlying processing mechanisms. To set the stage for a demonstration of how SDA works, we will first discuss established modelling approaches for analysing RTs.

## Process and measurement models

There are two main types of models that can be distinguished in the analysis of RT distributions: process models and measurement models (Anders, Alario, & Van Maanen, 2016; Howard et al., 2023). In process models, the emphasis is on making explicit the processes that give rise to observed RT distributions, as in the drift-diffusion model (Ratcliff et al., 2004; Ratcliff et al., 2016), the LATER (Carpenter 1981; Noorani & Carpenter, 2016) and the E-LATER models (Nakahara, Nakamura, & Hikosaka, 2006), the linear ballistic accumulator model (Brown & Heathcote, 2008), interactive activation models (McClelland & Rumelhart, 1981; Rumelhart & McClelland, 1982), the spatial coding model (Davis, 2010), and the Bayesian reader (Norris, 2006) (for a discussion, see Norris, 2013; Luce, 1986). For example, the drift-diffusion model assumes noisy integration of information until a threshold is reached. In contrast, measurement models describe the shape of RT distributions by directly fitting probability distributions such as the Wald or ex-Gaussian distributions to measured RTs (Anders, Alario, & Van Maanen, 2016; Matzke & Wagenmakers, 2009). This approach is computationally cheaper than for process models, because it only requires the evaluation of simple functions such as an exponential function. When selecting which probability distributions to fit, analysts consider properties of the data; a key feature of RT distributions is their positive skew. While the Wald and the ex-Gaussian distributions are both positively skewed, they are not the only ones. For example, the gamma and lognormal (lnorm) distributions also exhibit positive skew and, therefore, could be good candidates for fitting observed RT distributions. Notably, since measurement models focus on the shape of the RT distribution, they are commonly considered to be purely descriptive, not explaining underlying processes.

A pivotal decision for any analyst is the choice of model. For process models, selection is often based on the mechanism being described and on computational costs. For instance, a drift-diffusion model is preferred when the emphasis is on a high-level description of noisy information integration and on keeping computational costs relatively low. On the other hand, an interactive activation model provides more description of the mechanisms that generate RTs but is also more complex and hence computationally more demanding due to the large number of variables that need to be computed. For measurement models, computational costs do not clearly distinguish between them, since most measurement models have the same or similar number of parameters, and it is this number that determines the computational load (but see Howard et al., 2023, where a different parametrisation of the Wald distribution was used to reduce computational demand by replacing sampling from a probability distribution with a simple function).

The described distinctions between measurement and process models suggest that these are fundamentally different approaches. However, this is too restrictive a notion. For example, the Wald distribution could be conceptualised as a distribution with two parameters, or the same distribution could be conceived as an evidence-accumulator model in which noisy information is integrated with a constant rate until it reaches a threshold (Anders, Alario, & Van Maanen, 2016; Howard et al., 2023; Steingroever, Wabersich, & Wagenmakers 2021). Put differently, the Wald distribution can be used for both a measurement and process model. Similar correspondences exist for other probability distributions. A gamma distribution describes the statistics of a sum of exponentially distributed random variables, which can be identified with serial information processing where each stage has an exponentially distributed execution time (Berman, 1981). The lnorm distribution is often employed when describing the product of independently and identically distributed positive random variables (Limpert, Stahel, & Abbt, 2001) and has been used to characterise RTs in a variety of tasks (De Boeck & Jeon, 2019; van der Linden, 2006).

Capitalising on the link between measurement models and process models is a promising approach for model selection because many models within the class of measurement models can be characterised based on a *mechanism*. These mechanisms range from low-level abstract descriptions such as serial processing to more concrete cognitive processes such as neural integration of information. Crucially, if the research goal is to distinguish between different measurement models, the mechanisms by themselves should not be used for model selection. In this case, measurement models are compared for their goodness of fit and, once a model is selected, it points to the underlying mechanisms. For example, if a gamma distribution is shown to best explain the data, this would strongly suggest an underlying serial process. Thus, the key focus when choosing a measurement model is to assess the goodness-of-fit relative to other distributions and select the one that is most consistent with the data. Finally, assessing measurement models and selecting the best fit can help identify theories that may account for the generation of RTs. However, to date, such model comparisons are extremely rare for measurement models (for somewhat of an exception see the work by Matzke & Wagenmakers, 2009).

Irrespective of the chosen model, one of the primary goals for investigating RT distributions is to characterise the processing of words and how word properties affect this. In particular, RT distributions contain words that differ on numerous characteristics such as word frequency and word length, and we would expect a model to reflect these fundamental properties. When fitting a process model, these properties can be directly incorporated into the parameters. For instance, word frequency modulates the resting level activation (RLA) in interactive activation models (McClelland & Rumelhart, 1981; Rumelhart & McClelland, 1982) or the drift rate in the drift-diffusion model (Wagenmakers, Ratcliff, Gomez, & McKoon, 2008), with more frequent words associated with higher values of RLA and drift rate, respectively. For measurement models, fitting all RTs of a single participant yields participant-specific parameter values (Yap et al., 2012). However, such fitting does *not* provide any information on how word properties like frequency and length shape these parameters. Thus, despite the computational advantages of measurement models over process models, the former fail to incorporate word properties in parameter estimates, making them potentially less attractive. This view appears to be supported in a study by Matzke and Wagenmakers (2009), where frequency effects were at best unclear when ex-Gaussian and Wald distributions were fitted to RTs of high-frequency, low-frequency, and very low-frequency words. More generally, a lack of explanatory power might render fitting observed RTs with known distributions such as the Wald or lnorm less attractive, even though it may be conceptually straightforward.

## Stratified distributional analysis (SDA)

The SDA proposed in the current study combines the computational and conceptual ease of measurement models while allowing us to gain insight into the mechanisms that generate RTs. SDA achieves this “best of both worlds” by applying hierarchical Bayesian modelling to the millions of lexical decision RTs available in two mega studies: the English Lexicon Project (ELP; Balota et al., 2007) and the British Lexicon Project (BLP; Keuleers, Lacey, Rastle, & Brysbaert, 2012). In addition, the development of SDA provides a framework for model comparison and model selection, thus empowering analysts to quantitatively distinguish between different probability distributions, which in turn can differentiate between different processes. Further, as will become apparent, SDA quantifies the impact of item-specific information, such as word frequency and word length, on the distributional parameters of measurement models for RT distributions. This will allow analysts to investigate differences in RT distributions between individual participants as a function of word properties. At the level of a single participant, coverage of word properties is usually sparse. As an example, consider word frequency and word length. Although participants see thousands of items, only a few of them have the same length and a comparable frequency. Thus, a naïve statistical model would be severely underpowered to arrive at any meaningful conclusion. The hierarchical Bayesian framework allows us to fill in the gaps that exist at the single-participant level by leveraging information contained in the entire dataset. In other words, we can learn about the responses of a single participant by investigating how other participants respond.

Note that SDA does not make any assumptions about how the distributional parameters of the measurement models scale as a function of word properties. For instance, in mixed effects models of any flavour (e.g., ordinary, generalised, or generalised additive), it is often assumed that the mean RT (or mean log RT) across participants depends on the logarithm of the word frequency, word length, neighbourhood size, and other item properties in a linear manner, potentially with interactions between the different properties. Such assumptions necessarily introduce a modelling bias. Crucially, SDA is sufficiently flexible to yield meaningful results without having to introduce such biases.

In addition, we will illustrate how SDA can be used both qualitatively and quantitatively to assess and differentiate between various measurement models—that is, SDA will provide analysts with a framework to contrast the performance of different measurement models in a controlled manner. Such comparisons will be essential to support or rule out commonly used measurement models (e.g., Wald, ex-Gaussian, gamma, lnorm), thus providing analysts with an informed choice for their future investigation of RT distributions.

## SDA and word frequency

While our SDA can be applied to any word characteristic, such as frequency of occurrence, word length, age of acquisition, or semantic neighbourhood, here we focus on word frequency, which will be examined at a range of word lengths. According to Whaley (1978), of all the variables that might influence word recognition, word frequency is by far the most powerful predictor of response times. Although the locus of word frequency effects has been disputed (e.g., Monsell, 1991; Murray & Forster, 2004), there is broad agreement that frequency impacts the structure of the lexicon (Brysbaert et al., 2011; Brysbaert, Mandera, & Keuleers, 2018; Rayner, 1998, 2009; for deviating views see Balota & Chumbley, 1984; Besner & Smith, 1992; Morrison & Ellis, 1995).

Frequency measures from corpora are often viewed as indexes of linguistic experience. However, corpora only provide an approximation of actual exposure, which may be particularly problematic for low-frequency words (Gernsbacher, 1984; Gardner et al., 1987). Although corpus frequency does not necessarily align with actual experience with a language, some corpora provide measures of frequency that explain more of the variance in response times. In particular, corpora that are based on subtitles from television and film outperform those based on written documents (Brysbaert & New, 2009; van Heuven, Mandera, Keuleers, & Brysbaert, 2014), as do those from social media (Herdağdelen & Marelli, 2017).

In addition to the size and the register of the corpus, how word occurrence is quantified is thought to be important (Brysbaert & New, 2009). A common and widespread approach is based on word form frequency, which counts the number of times a word occurs in a corpus, see the Kučera and Francis corpus (1967), CELEX (Baayen et al., 1993), the BNC (2007), SUBTLEX-UK (van Heuven et al., 2014), and SUBTLEX-US (Brysbaert & New, 2009). An alternative measure—contextual diversity—provides a different quantification of word occurrence that accounts for the number of contexts in which a word is encountered (Adelman, Brown, & Quesada, 2006). Contextual diversity is distinct from word form frequency in that its impact on RTs is thought to be mediated through a different mechanism than that for word form frequency (Adelman, Brown, & Quesada, 2006; Perea et al., 2013; Vergara-Martínez et al., 2017). Recently, Johns (2021) developed discourse contextual diversity (DCD) and user contextual diversity (UCD) based on an analysis of over 55 billion words from the internet platform Reddit. The debate as to what is the most appropriate quantification of word occurrence is ongoing (for a recent account, see Gries, 2022). For the present study, we employed DCD, UCD, and word form frequency counts from the SUBTLEX-US data. For ease of discussion, we will generally refer to all three measures as word frequency but emphasise the differences between them as and when needed.

Given that the present research introduces SDA for the first time, it is fitting to show its workings by applying it to word frequency as a key predictor of RTs. As we will demonstrate, SDA allows us to evaluate and compare different frequency measures (i.e., subtitle frequency vs. contextual diversity), which is important as it may point towards the most appropriate measure of how to quantify word frequency. Crucially, SDA reveals how word frequencies contribute to the composition of RT distributions, which opens up the possibility of probabilistically predicting RTs when properties of a word are known (e.g., length and frequency). This prediction will be accomplished at the single-participant level since the Bayesian estimation of SDA will provide posterior distributions for all parameters for individual participants. These estimates of single-participant parameter values pave the way for dissecting the performance of individual participants, potentially highlighting processing difficulties and their origins. For instance, does a particular participant struggle with all low-frequency words, or are some word frequencies more problematic than others? If RTs are generally slow, is this because of issues with information processing or because of slow motor responses (or a combination of both)? While an overall RT distribution might look reasonable, SDA reveals details within it and hence provides a high-resolution diagnostic tool for the study of RT distributions.

## Methods

### Response time and word-frequency data

We will show the step-by-step application of SDA based on the lexical items in the English Lexicon Project (ELP, Balota et al., 2007) and the British Lexicon Project (BLP, Keuleers, Lacey, Rastle, & Brysbaert, 2012). The ELP contains behavioural data from visual lexical decision and word naming for 40,481 American English words and 40,481 nonwords. A total of 815 subjects participated in the first task, while 443 subjects took part in the second task. For the current work, only the lexical decision data is used. In the BLP, 78 participants were split into two groups, each responding to a visual lexical decision on 14,365 British English monosyllabic and disyllabic words and an equal number of nonwords. The single trial data from the two databases, which are analysed, are available at https://osf.io/n63s2/ (ELP) and https://osf.io/b5sdk/ (BLP). As instructed on the Wiki page accompanying the OSF archive for the ELP, we excluded the files 9999.LDT, 793DATA.LDT, Data999.LDT, Data1000.LDT, Data1010.LDT, and Data1016.LDT. Next, only participants with an accuracy of at least 60% were included, which resulted in three participants (IDs: 576, 791, 696) being removed. Then, we computed the percentage of RTs for each participant that were faster than 150 ms and slower than 2,000 ms. If this was larger than 20%, the participant was excluded from the analysis. This step resulted in eight participants being removed (IDs: 169, 180, 183, 222, 292, 27, 62, 532). Overall, the preprocessing of the ELP single-trial data resulted in 11 participants being excluded. For the BLP, we followed the same preprocessing steps, which resulted in no participants being removed from the analysis.

The analysis looks at two frequency measures. A word frequency measure was based on SUBTLEX-US (Brysbaert and New, 2009), a database of 51 million words that contains word frequencies from the subtitles of English-US movies and TV series. The second was a contextual diversity measure that was obtained from over 55 billion words from the internet platform Reddit (Johns, 2021). Only words for which both RT and word frequency were available were included in the analysis. In addition, we only considered words having a length between 4 and 10 characters and excluded words with the part-of-speech (POS) “minor” for the ELP and “Interjection,” “Conjunction,” “Pronoun,” “Numera,” “Preposition,” “Interjection,” “Article,” and “Undefined” for the BLP. This resulted in 778,191 items for the ELP and 717,763 items for the BLP.

### Hierarchical Bayesian model

**Figure 1.**
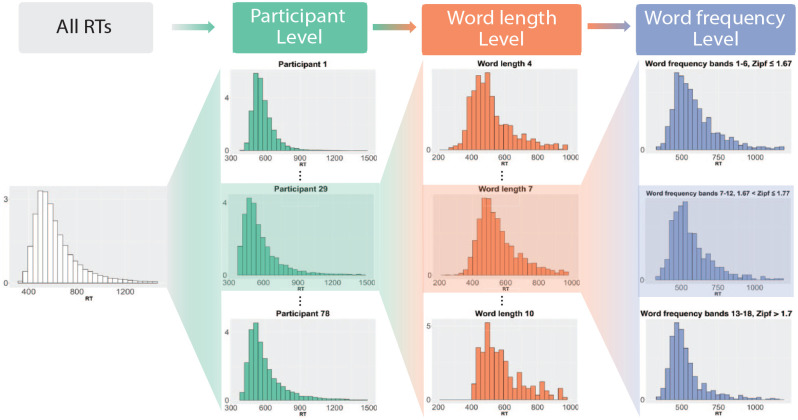
Schematic of the stratification employed in the analysis. The depicted distributions are based on data from the BLP.

Figure 1 contains a schematic of the stratification that we employed for SDA. The RTs of all participants are at the top level (left-hand side). The next level is constituted by RTs from individual participants, which in turn are split along word length and word-frequency bands in the next two levels of the hierarchy. We illustrate this procedure in more detail with participant 29. Note that for illustrative purposes, we only show three levels for word length (4-, 7-, and 10-character words) and have lumped word-frequency bands together. In the analysis, word-frequency bands group items together with similar frequencies. In the past, such groupings were employed to study word-frequency effects. For example, Matzke and Wagenmakers (2009) created three word-frequency bands (high frequency, low frequency, and very low frequency), while Yap et al. (2008) had seven vincentiles/bands. In the present study, we employed a much more fine-grained representation of word frequency. Words were divided into frequency bands such that each contained at least 40,000 observations. This number was chosen to ensure sufficient data in each frequency band for the analysis. To obtain the frequency bands, quantiles of the word frequencies were computed, which ensured that no word frequencies belonged to two different frequency bands. Put differently, word frequencies were uniquely associated with a word-frequency band. Using quantiles means that different word-frequency bands may have different numbers of observations and that the bands differ between the ELP and BLP. Overall, there were 20 bands for the ELP and 18 bands for the BLP.

The structure of the stratification entails that each RT is represented as a variable with four indices: $Y_{ijkl}$ , where j denotes the participant ID, k refers to the word-frequency band, l indexes the word length, and i enumerates the RT. Each RT is decomposed into a deterministic and stochastic component such that

(1) $$Y_{ijkl}=\theta_{j}+X_{ijkl}.$$

This was motivated by the insight that RTs contain an “irreducible minimum” (Rouder et al., 2005, p. 205) or a time “where the respondent cannot appropriately complete the task this quickly” (Anders, Alario, & Van Maanen, 2016, p. 2). In Equation (1) the deterministic component $\theta_{j}$ subsumes motor functions such as pressing a response key. Notably, $\theta_{j}$ is independent from item properties but subject-specific. In contrast, the integration of word properties is noisy and depends on item properties. In short, we assume that each participant has their own deterministic component $\theta_{j}$ and that item properties such as word frequency and word length shape the stochastic contribution (hence the additional indices k and l for $X_{ijkl}$ ). In the analysis, the stochastic contribution $X_{ijkl}$ is modelled using the lnorm, Wald, and gamma distributions to ascertain which best accounts for the data. Since $\theta_{j}$ is constant, the probability distribution for $Y_{ijkl}$ follows directly from that of $X_{ijkl}$ :

(2) $$f_{\mathrm{lnorm}}\left(y_{ijkl}|\theta_{j},\rho_{jkl}\right)=\frac{1}{\sqrt{2\;\pi \sigma_{jkl}^{2}{\left(y_{ijkl}-\;\theta_{j}\right)}^{2}}}\exp\left(-\frac{{\left(\ln\;(y_{ijkl}-\;\theta_{j})-\mu_{jkl}\right)}^{2}}{2\sigma_{jkl}^{2}}\right),$$

(3) $$f_{\mathrm{Wald}}\left(y_{ijkl}|\theta_{j},\rho_{jkl}\right)=\sqrt{\frac{\lambda_{jkl}}{2\pi {\left(y_{ijkl}-\theta_{j}\right)}^{3}}}\exp\left(\frac{-\lambda_{jkl}{\left(y_{ijkl}-\theta_{j}-\mu_{jkl}\right)}^{2}}{2\mu_{jkl}^{2}(y_{ijkl}-\theta_{j})}\right),$$

(4) $$f_{\mathrm{Gamma}}\left(y_{ijkl}|\theta_{j},\rho_{jkl}\right)=\frac{\beta_{jkl}^{\alpha_{jkl}}}{\Gamma \left(\alpha_{jkl}\right)}{\left(y_{ijkl}-\theta_{j}\right)}^{\alpha_{jkl}-1}e^{-\beta_{jkl}(y_{ijkl}-\theta_{j})}.$$

The distributions in Equations (2)–(4) are known as shifted distributions since they depend on the difference between $y_{ijkl}$ and $\theta_{j}$ —that is, $y_{ijkl}-\theta_{j}$ (Anders, Alario, & Van Maanen, 2016; Howard et al., 2023; Matzke & Wagenmakers, 2009; Steingroever, Wabersich, & Wagenmakers 2021). To make this shift explicit, we split the parameters for each distribution into the shift, which is the deterministic contribution $\theta_{j}$ , and the remaining parameters. This yields $\rho_{jkl}=\{\mu_{jkl},\sigma_{jkl}^{2}\}$ for the lnorm, $\rho_{jkl}=\{\mu_{jkl},\lambda_{jkl}\}$ for the Wald, and $\rho_{jkl}=\{\alpha_{jkl},\beta_{jkl}\}$ for the gamma distribution. The introduction of $\rho_{jkl}$ is for presentational convenience only. It allows us to refer to those parameters of the distribution that are not the shift $\theta_{j}$ without having to provide details of the distribution.

Since $\rho_{jkl}$ subsumes two parameters for the lnorm, Wald, and gamma distributions, we can refer to them as $\rho_{jkl}^{\left[1\right]}$ and $\rho_{jkl}^{\left[2\right]}$ . To set up the hierarchical Bayesian estimation (Anders et al., 2018; Schad et al., 2023; Shiffrin et al., 2008), we require priors for these parameters. As $\rho_{jkl}^{\left[1\right]}$ and $\rho_{jkl}^{\left[2\right]}$ are positive for the three distributions in (2)–(4), the priors’ distributions should be zero for negative values and nonzero for positive values. The gamma distribution satisfies these constraints, and so we set the priors as

(5) $$\rho_{jkl}^{\left[1\right]}∼\mathrm{Gamma}\left(\alpha_{l}^{\left[1\right]},\beta_{l}^{\left[1\right]}\right),\rho_{jkl}^{\left[2\right]}∼\mathrm{Gamma}\left(\alpha_{l}^{\left[2\right]},\beta_{l}^{\left[2\right]}\right).$$

The parameters $\alpha_{l}^{\left[1\right]},\beta_{l}^{\left[1\right]},\alpha_{l}^{\left[2\right]},\beta_{l}^{\left[2\right]}$ only depend on l , which means that words with different lengths have different priors.

Turning to the deterministic contribution, $\theta_{j}$ is positive. Following the same argument as above, we employ a gamma distribution as a prior:

(6) $$\theta_{j}∼\mathrm{Gamma}\left(\alpha_{\theta },\beta_{\theta }\right).$$

Thus far, the priors have been characterised by the parameters $\psi =\left(\alpha_{l}^{\left[1\right]},\beta_{l}^{\left[1\right]},\alpha_{l}^{\left[2\right]},\beta_{l}^{\left[2\right]},\alpha_{\theta },\beta_{\theta }\right)$ . Because we consider words with lengths between 4 and 10 characters (i.e., seven word lengths), there are a total of 30 parameters in ψ : seven $\alpha_{l}^{\left[1\right]}$ , seven $\beta_{l}^{\left[1\right]}$ , etc., plus $\alpha_{\theta }$ and $\beta_{\theta }$ for the deterministic contribution. Following hierarchical Bayesian modelling, priors are required for the 30 parameters in the set ψ . As these parameters describe the shape and rate of a gamma distribution, they are positive. Consequently, we again use a gamma distribution, that is,

(7) $$\phi_{i}∼\mathrm{Gamma}\left(a_{i},b_{i}\right),\phi_{i}\in \psi ,$$

where $a_{i},b_{i}$ , were chosen before carrying out the analysis. Putting everything together, 30 parameters $a_{i}$ and $b_{i}$ were chosen, which determine the α ’s and β ’s in the set ψ, which in turn shape the $\rho_{jkl}$ and $\theta_{j}$ .

### Model assessment

To assess the models, we employ posterior predictive checking, which involves sampling data from the posterior predictive distribution and comparing it with the observed data. If the model fits well, the sampled data should look similar to the observed data (Gelman et al., 2013). The posterior predictive distribution is then given by

(8) π(z|y)=∫f(z|ψ)π(ψ|y)dψ.

In Equation (8), π(ψ|y) and f(z|ψ) denote the posterior density and sampling density, respectively. The y is the collection of all RTs and f is any of the probability distributions under investigation, such as the shifted lnorm distribution in Equation (2).

Comparing probability distributions directly can be challenging, especially for high-dimensional data, like that in the ELP and BLP. A common solution is to compare summary statistics instead, such as the mean of a probability distribution. We adopt this approach and compare the mean of the posterior predictive distribution in Equation (8) with the mean of the data. Due to the hierarchical framework, means need to be compared across participants, word lengths, and word-frequency bands. To quantify how different the observed means are from the computed ones, the posterior predictive *p*-values are computed (for more details, see Gelman et al., 2013, chapter 6), which can be seen as the Bayesian analogue of traditional *p*-values used in frequentist approaches. In general, posterior predictive *p*-values close to 0.5 indicate good model fits, whereas values close to 0 or 1 indicate poor model fits. A comprehensive view of posterior predictive checking for hierarchical models can be found in Sinharay and Stern (2003).

### Model selection

While model assessment aims at verifying the validity of underlying model assumptions, model comparison seeks to directly compare a series of competing models. To select models in the present work, we employed the expected log pointwise predictive density (elpd), which is defined as (Vehtari et al., 2017)

(9) $$\mathrm{elpd}=\sum_{i=1}^{n}\int \;p_{t}\left(\tilde{y_{i}}\right)\log\pi (\tilde{y_{i}}|y)d\tilde{y_{i}},$$

where $p_{t}\left(\tilde{y_{i}}\right)$ denotes the (unknown) true data-generating process for an unobserved new observation $\tilde{y_{i}}$ , and $\pi (\tilde{y_{i}}|y)$ is the posterior predictive distribution defined in Equation (8). In practice, we must approximate Equation (9) since the true sampling density is unknown.

Models can be ranked using pairwise comparisons by estimating the difference in elpd between them. In the current research, instead of reporting all pairwise comparisons, we used the optimal model as the reference model and looked at all elpd differences relative to the reference model.

## Results

The findings are presented in two parts. We begin by illustrating the information-rich results that the hierarchical Bayesian model yields, followed by model assessment and selection. For the Bayesian model, we show posteriors of distributional parameters and how these parameters vary as a function of word frequency and word length. In the model assessment, we demonstrate that SDA yields good agreement between measured and modelled RT distributions for both the BLP and ELP as quantified by posterior predictive distributions and their *p*-values. Further, we demonstrate that model selection can be achieved via expected log predictive densities, which identify the lnorm distribution as the probability distribution that is most consistent with the data.

**Figure 2.**
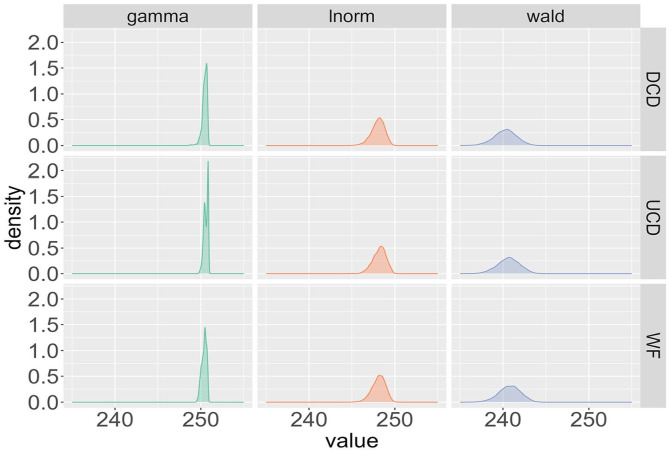
Posterior distributions of the deterministic contribution θj for participant 29 from the BLP across the three different probability distributions (Wald, gamma, lnorm) and the three frequency measures (WF, UCD, DCD).

The starting point of our analysis examines the deterministic contribution $\theta_{j}$ to RTs (see Equation (1)). Figure 2 shows the posterior distributions of $\theta_{j}$ for a single participant across the three probability distributions (Wald, gamma, lnorm) and the three frequency measures (WF, UCD, DCD) that were tested. For each of the three distributions, the posteriors look similar irrespective of the word-frequency measure. For instance, in Figure 2, the three panels depicting the Wald distribution are all centred around a mean of 240 ms and exhibit a similar standard deviation.

**Figure 3.**
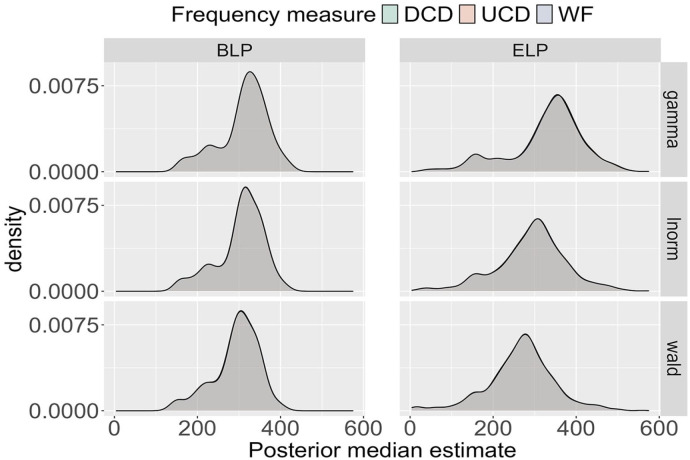
Posterior median for the deterministic θj across all participants in the BLP and ELP for the three probability distributions (Wald, gamma, lnorm) and the three frequency measures (WF, UCD, DCD). Note that the distributions are visibly indistinguishable for a given probability distribution and data source.

To compare the deterministic $\theta_{j}$ across participants, the medians of the posterior distributions were computed for each participant. The results for all participants are displayed in Figure 3, which contrasts the findings for the BLP (left) with those for the ELP (right). While all three frequency measures were tested, the results are indistinguishable in the figure. Thus, only one distribution of medians is visible in each panel.

In Figure 3, for the BLP, the shape of the median distributions is the same across the three probability distributions, with the only difference being a small horizontal shift. More specifically, the results for the Wald distribution are shifted slightly leftwards, reflecting shorter RTs compared to those of the other distributions. The ELP results exhibit more variability. The shape of the median distribution varies from the gamma to the lnorm to the Wald distribution as evidenced by the presence of a minor second peak for smaller values of the median for the gamma distribution, which is absent for the lnorm and Wald distributions. In addition, horizontal shifts are more pronounced for the ELP than for the BLP. Despite these variations, the estimates of the medians for both the BLP and the ELP are consistent with previously reported nondecision times, which correspond to the deterministic contribution of RTs (e.g., Anders, Alario, & Van Maanen, 2016).

While the median distributions for the ELP vary to some extent, the variations are rather small. Coupled with the consistent shape of the median distribution for the BLP, these findings suggest that the deterministic contribution $\theta_{j}$ is independent of the stochastic component $X_{ijkl}$ (see Equation (1)). Put differently, if the choice of the distribution for the stochastic part—that is, the Wald, gamma, or lnorm distribution, impacted the estimate for the deterministic contribution $\theta_{j}$ , the estimates for $\theta_{j}$ should be different for the three different probability distributions. Since this is not the case, we can infer that $\theta_{j}$ and $X_{ijkl}$ are conceptually independent. This is consistent with our intuition that the deterministic contribution, which governs, for instance, visual encoding and motor movement, should not influence the information processing that is described by the stochastic component.

Finally, comparing the application of SDA to the BLP and ELP makes it evident that the distributions in the BLP are shifted towards the left. Given that RTs from the BLP are generally faster than those from the ELP, this difference can be partially attributed to a faster nondecision time for the BLP participants. Hence, SDA helps to quantify intrinsic differences between the BLP and ELP.

**Figure 4.**
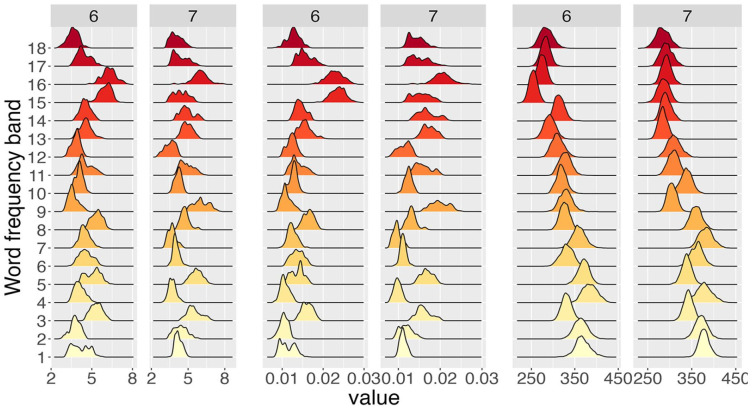
Posterior distributions for the shape α (left two columns) and the rate β (centre two columns) of the gamma distribution together with the posterior distributions for the mean α/β (right two columns) across word-frequency bands (computed from WF) for six- and seven-character words for participant 29 from the BLP.

Having estimated the deterministic contribution $\theta_{j}$ , we now turn to the stochastic component $X_{ijkl}$ . To illustrate the kind of information-rich results that our hierarchical Bayesian analysis yields, we use the gamma distribution as an example and restrict word lengths to a few values. Figure 4 displays the posterior distributions for the shape α , the rate β , and the mean α/β of the gamma distribution for words that are six and seven characters long for a single participant. An initial visual inspection reveals that there is a clear trend for the mean (last two columns in Figure 4): the distributions shift towards the left with increasing word-frequency bands. While there is some rightward movement of the distributions for β with increasing word-frequency bands, the distributions for α show no clear tendency. However, combining the results for α and β allows us to understand the behaviour of the mean α/β . The increase of β with increasing word frequency is responsible for the decrease of α/β with increasing word frequency, and α does not offset the influence of β . Crucially, we can further interpret the results for α and β . Since β governs the exponential tail of the gamma distribution (see Equation (4)), an increase of β with increasing word frequency results in increasingly shorter tails. Together with the results for α , this shorter tail leads to overall shorter RTs, as would be expected for words with larger word frequencies.

**Figure 5.**
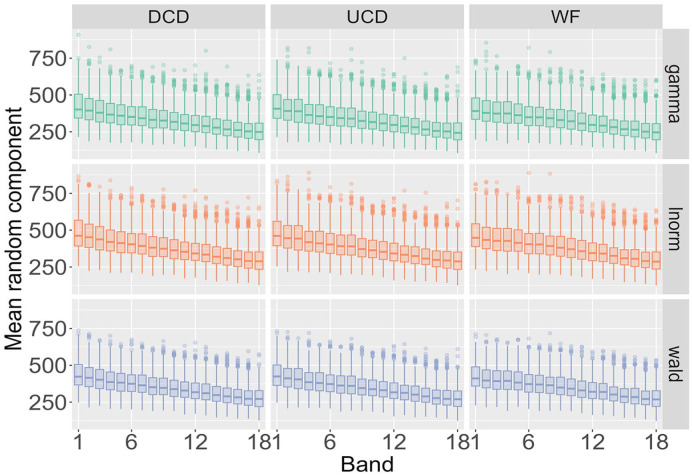
Posterior distribution for the mean of the random component X_ijkl_ across the three probability distributions and three frequency measures as a function of word frequency for the BLP.

Figure 4 highlights the type of detailed information about a single participant that our analysis yields and helps to map out how RT distributions change as a function of word frequency and word length. However, the findings need to be compared across all participants, data sources (BLP vs ELP), the three probability distributions (Wald, gamma, lnorm), and the three frequency measures (WF, DCD, and UCD). Figure 5 displays such a comparison for the mean of the distributions across the 9 combinations of the three probability distributions and three frequency measures when data is amalgamated over word length to show the impact of word frequency. As done previously for the deterministic contribution $\theta_{j}$ (Figure 3), the single participant distributions as illustrated in Figure 4 are summarised by their means across all participants. For the 9 combinations shown in Figure 5, the posterior distributions decrease with increasing word frequency. Moreover, the results are consistent across the 9 combinations—that is, the box plots span the same range of values for a given word-frequency band irrespective of the combination of probability distribution and frequency measure. This points towards a robust estimation of the parameters of the probability distributions since the mean values reported in Figure 5 are computed from the individual parameters of the distributions (in the same manner as was illustrated previously for the gamma distribution, where the mean α/β was computed from the two parameters *α* and *β*).

**Figure 6.**
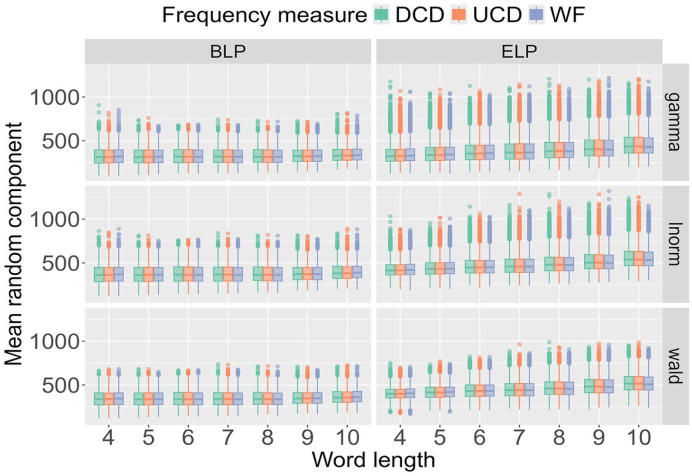
Posterior distribution for the mean of the random component X_ijkl_ across the three probability distributions and three frequency measures as a function of word length.

To understand the impact of word length, the results in Figure 6 are amalgamated over word frequency. For both the BLP and ELP, the results are almost identical for the three frequency measures. In other words, when fixing a probability distribution and a word length, the results do not differ depending on how word frequency is quantified. There is, however, a crucial difference between the BLP and ELP. For the BLP, word length only marginally impacts the results, while the estimates for the ELP increase as a function of word frequency.

Figures 2–6 illustrate the information-rich results that the hierarchical Bayesian model yields, revealing how parameters of the RT distributions scale as a function of word length and word frequency at both the single-participant level and across all participants.

**Figure 7.**
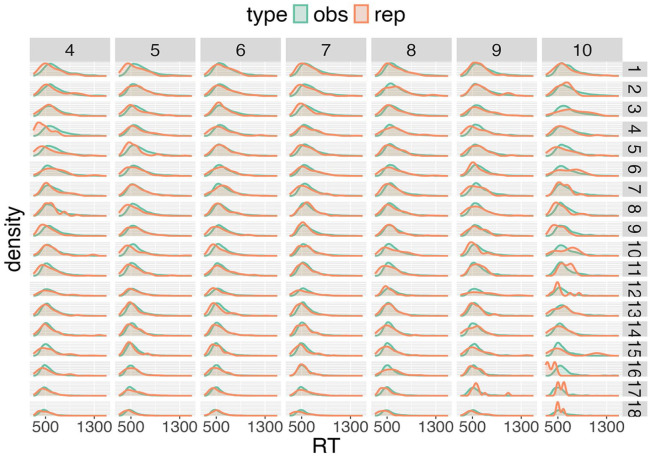
Posterior predictive distributions for participant 29 of the BLP for the gamma distribution.

What is needed next is to compare the fitted RT distributions to the measured RTs, which is achieved by model assessment via posterior predictive distributions and then model selection based on expected log predictive densities. In Figure 7, the posterior predictive distribution for a single participant is shown together with the measured RTs. Overall, excellent agreement between the estimated probability distributions and the experimental RTs can be observed. The few discrepancies that occur generally result from sparse observations for a participant—for example, RTs for long, high-frequency words are less represented in the dataset.

**Figure 8.**
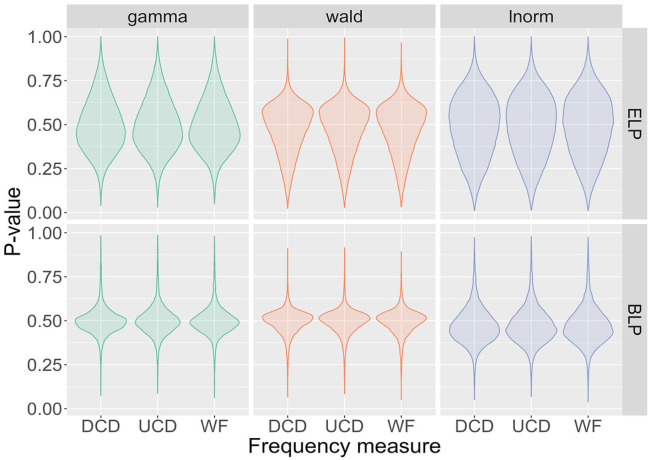
Violin plots of posterior predictive *p*-values for all 18 models. The distributions are centred around 0.5, indicating that the models are consistent with the data.

In the next step, we quantify how well the posterior predictive distributions describe the measured data. For this, posterior predictive *p*-values (ppp-values) were computed, with the results displayed in Figure 8. The ppp-values for most models are centred around 0.5. This indicates that the fitted models are consistent with the measured data, since ppp-values close to 0.5 indicate good model fits, whereas values close to 0 or 1 indicate poor model fits (Sinharay & Stern, 2003). On closer inspection, the distribution of ppp-values is more condensed for the BLP than for the ELP. This reflects the larger variation amongst participant in the latter database. It is also noticeable that results cluster for a given distribution. For example, the mean of the ppp-value distributions is around 0.5 for the gamma distribution but lower than that for the Wald distribution and higher than that for the lnorm distribution.

With ppp-values around 0.5, the results in Figure 8 demonstrate that all models are consistent with the data—that is, none of the models can be considered to provide a poor representation of the data. This leaves us with the question of which model best explains the data. Table 1 lists the differences in expected log predictive densities (elpd) for the different models. For each lexicon project, the model with the smallest elpd is considered to be the best and is shown with a value of 0 in the Δ elpd column in Table 1. Next, the difference in elpd between the best model and the other models is computed, and the models are ranked by increasing elpd difference. For both the BLP and the ELP, the model with the lnorm distribution is identified as the most consistent with the data, with DCD and UCD being the best frequency measure for the BLP and ELP, respectively. Note, however, that the standard error associated with the second-best model (UCD for the BLP, DCD for the ELP) is larger than the estimated difference. This indicates that the reported difference between UCD and DCD could be due to chance and not statistically significant. While we cannot clearly distinguish between the goodness of the UCD and DCD, using WF always performs worst, as revealed by the large elpd difference.

**Table 1.** Difference in expected log-predictive density ( Δ elpd) and standard error (se) for all models studied. Comparisons are performed separately for each data source (BLP, ELP).

BLP			ELP		
Model	Δe lpd	se	Model	Δ elpd	se
DCD_lnorm	0.00	0.00	UCD_lnorm	0.00	0.00
UCD_lnorm	–3.42	165.55	DCD_lnorm	–276.17	308.41
WF_lnorm	–3304.10	248.90	WF_lnorm	–1205.45	369.11
DCD_wald	–66356.97	363.39	UCD_wald	–29293.34	390.23
UCD_wald	–66382.14	382.59	DCD_wald	–29706.60	441.88
WF_wald	–69637.79	405.86	WF_wald	–31413.95	461.44
WF_gamma	–1436103.28	1337.66	WF_gamma	–1494170.78	1328.28
UCD_gamma	–1444745.31	1335.30	DCD_gamma	–1494835.56	1313.71
DCD_gamma	–1448043.07	1326.09	UCD_gamma	–1500317.07	1289.44

## Discussion

Measuring and analysing RTs has been extremely fruitful in furthering our understanding of word recognition (Luce, 1986). While early approaches mainly focused on summary statistics such as mean RTs, RT distributions have now taken centre stage (Balota, Yap, Cortese, & Watson, 2008; Balota & Yap, 2011; Lo & Andrews, 2015; Rieger & Miller, 2020). In the present research, we developed SDA, or stratified distributional analysis, to study RT distributions as a function of independent variables such as word frequency and word length. We showed that RT distributions obtained from two independent megastudies (ELP: Balota et al., 2007; BLP: Keuleers, Lacey, Rastle, & Brysbaert, 2012) are most consistently described by an lnorm distribution as a function of word frequency and length, when word frequency was quantified using contextual diversity. When fitting RT distributions across word-frequency bands using a hierarchical Bayesian framework, we obtained estimates of the parameters of the RT distributions together with a measure of their uncertainty as a function of word frequency and word length. Crucially, these estimates quantified how words with different frequencies determine the probability of finding a given RT. We also provided estimates for the deterministic times $\theta_{j}$ in the lexical decision tasks. Importantly, these estimates are in good agreement with previously reported values and were obtained using uninformative priors for $\theta_{j}$ ; that is, we made minimal assumptions on the range of the $\theta_{j}.$

SDA was designed to answer several theoretically important questions. Firstly, given individual RT distributions, what is the probability distribution that most consistently describes the data, and does this differ depending on how word frequency is quantified? Secondly, can the deterministic part $\theta_{j}$ of RTs be reliably estimated, and are the estimates consistent with previous findings? In what follows, we will answer these questions before discussing extensions of our work.

To address the first question, we fit probability distributions with simple expressions (such as the Wald, gamma, and lnorm distributions) rather than simulating process models that describe high-level noisy integration of information (e.g., the drift-diffusion model) or are based on complex networks (e.g., interactive activation models). This choice was motivated by computational considerations. Fitting probability distributions generally requires fewer computational resources than simulating and fitting process models for which simple expressions are not available. It might appear that by fitting probability distributions we lose mechanistic insight. However, in the introduction, we discuss how the distributions tested in the present study are linked to specific processes. Thus, while SDA was performed on simple probability distributions, the results can be interpreted with respect to cognitive processes.

Our analysis demonstrated that the lnorm distribution is most consistent with the data. The lnorm distribution is often employed when describing the product of independently distributed positive random variables (Limpert, Stahel, & Abbt, 2001) and implies a sum of random log-transformed variables, which suggests a multistep process. This is consistent with the view that lexical decision involves a high-level structure: an initial parallel and automatic activation of lexical possibilities followed by serial and nonautomatic decision processes that lead to a response (Dijkstra & Peeters, 2023). The lnorm distribution has been previously reported to fit RT distributions (Luce, 1986; Rouder, 2005), while more recently, the Wald distribution has been used in the analyses of RTs (Anders, Alario, & Van Maanen, 2016; Howard et al., 2023; Matzke & Wagenmakers, 2009; Steingroever, Wabersich, & Wagenmakers 2021). In these studies, the use of the Wald distribution is motivated by the link to noisy integration processes. Since the Wald distribution was the second-best model in our analysis, it would be instructive to compare the published results with a re-analysis of the data using an lnorm distribution.

The link between the lnorm distribution and the product of independently distributed positive random variables illustrates how simple probability distributions can emerge from process models. This relationship, however, is not unique (McElreath, 2020). Multiple process models can give rise to the same measurement model––that is, the same probability distribution. For instance, the lnorm distribution also describes an evidence-accumulator model in which noisy information is integrated when both the drift rate and the noise strength are proportional to the level of evidence (Capocelli & Ricciardi, 1972). This many-to-one relationship renders SDA a powerful selector of potential process models and thus provides the basis for designing more detailed tests to distinguish between them, and in doing so, expands our insight into the governing processes.

A key component of SDA is the use of word-frequency bands. This approach allows us to determine the scaling of the distributional parameters without having to impose any specific form on the scaling. Indeed, it is not *a priori* obvious what scaling to choose. To illustrate this point, consider resting level activation in interaction activation models. While there is general agreement that resting level activation should increase with words of increasing frequency, the actual form of this scaling is still open to debate. For example, Dijkstra et al. (2019) employ two scalings involving a natural logarithm and a reciprocal-of-root function. By using frequency bands, we sidestep this debate.

The theoretical usefulness of word-frequency bands was also demonstrated in an investigation of word learning by Ellis et al. (2004), who split their test items into 100 word-frequency bands and randomly sampled words from them to obtain good frequency coverage and to allow for comparison between different languages. A notable difference between the work by Ellis et al. (2004) and ours is that instead of fixing the number of word-frequency bands *a priori*, the current word-frequency bands were designed to ensure a sufficient number of observations to reliably fit distributions (i.e., there were at least 40,000 observations per word-frequency band).

Another way of looking at bands—in this case, word-frequency bands—is that they reduce the variability of the variable being banded. From a modelling perspective, less variability is advantageous because it reduces the degrees of freedom that need to be accounted for in the model. In the current study, items within a word-frequency band had very similar frequencies, which reduces the variability of word frequency within the bands. In addition, by explicitly accounting for word length in SDA, any variability in length was effectively eliminated as well. If word frequency and word length were the only factors that shape RTs, the remaining variability in the model should come from the uncertainty in the measurement process, which is typically Gaussian. However, this was not the case, indicating that other factors are implicated in the description of RTs. Crucially, SDA can incorporate factors such as AoA or neighbourhood and hence may help to establish quantitative relationships between different factors.

Turning to the quantification of word frequency, a key finding in the present study is that contextual diversity measures perform best. There is consensus that words that occur more frequently have faster RTs than words with matched properties but fewer occurrences. However, how to best quantify word frequency has been debated. It has been shown that contextual diversity predicts RTs better than word form frequency and is distinct from it (Adelman et al., 2006; Perea et al., 2013; Vergara-Martínez et al., 2017), but other measures such as word prevalence (Brysbaert et al., 2016) have also been recently proposed as a determinant of RTs. It is important to note that word form frequencies are primarily based on the orthography and phonology of words, while contextual diversity takes into account semantics. The fact that SDA identified contextual diversity as being superior to word-form frequency lends further support to recent trends that RTs integrate more information than pure orthographic and phonological details.

In the analysis, we decomposed RTs into deterministic $\theta_{j}$ and stochastic components $X_{ijkl}$ . We assumed that $\theta_{j}$ could vary across participants—that is, each participant has their own $\theta_{j}$ . For a given participant, $\theta_{j}$ was the same across all word-frequency bands but allowed to be different for different frequency measures. We found that $\theta_{j}$ was independent of both the frequency measure and the probability distribution that describes the stochastic component $X_{ijkl}$ of the RTs. This aligns with the notion that there are certain processes in speeded RT experiments that are independent of an item’s properties, such as initiation and execution of the motor program to press a key. In that sense, our interpretation of $\theta_{j}$ corresponds with what Rouder et al. (2015) refer to as the “irreducible minimum” (see also Anders, Alario, & Van Maanen, 2016). It is important to point out that recent simulations and single-trial analysis of lexical decision RTs based on the drift diffusion model found that the deterministic component (often referred to as the nondecision time in these analyses) can vary with word frequency and accuracy (Dutilh et al., 2012, Gomez and Perea, 2014). An interesting future research avenue could extend SDA to allow for a word-frequency-dependent deterministic component—that is, instead of estimating $\theta_{j}$ , the model would depend on $\theta_{jk}$ .

At its heart, SDA provides a conceptual framework to organise and describe data. How to estimate the parameters of the resulting model is a different question. We opted for a hierarchical Bayesian framework, but a frequentist approach could have been used instead. A key advantage of a Bayesian approach over a frequentist one is that posterior distributions are obtained instead of merely yielding point estimates. Not only do posterior distributions help with quantifying uncertainty—and hence provide the analyst with a quantitative measure of how much they should believe the results—but they also allow for detailed model assessment and model selection. In the present study, models were assessed with ppp-values, which were calculated by looking at the proportion of simulated data sets that are greater than what was observed in the mean of the observed data. In other words, a single-point estimate was used to compare different distributions. In future work, more properties of the posterior distributions could be considered. This would allow for a more fine-grained comparison between the measured RT distributions and the posterior distributions, as well as indicating for which ranges of RTs the two distributions agree and exhibit notable differences.

While we have applied our approach to RTs from lexical decision tasks, our methodology is not task specific. We can analyse data from any experiment that generates RTs, irrespective of the task involved. For instance, our framework can be readily used for RTs from word naming. An interesting question is whether different tasks lead to different RT distributions. If so, this might point toward task-specific processes involved in the generation of RTs. On the other hand, similarities in the estimated RT distributions could be used to establish a common cognitive framework. Furthermore, our methodology can be applied to different item types and participant groups, again checking for differences and similarities in the fitted RT distributions. More specifically, SDA permits comparison of items within a single participant and could be used to identify how RTs for a set of items are related to RTs for another set of items. Regarding participants, a natural extension is to consider performance of varied participant groups like monolinguals and bilinguals, or young and older populations. In addition to RTs, reading times from eye-tracking could offer another application for our work. It would be interesting to explore whether the current framework holds for eye-tracking data involving the continuous reading of text, where context in the form of factors like predictability/surprisal impacts the reading time distributions. It is also important to point out that the current framework places no restrictions on the language under investigation. Our emphasis has been on American and British English (through the use of the ELP and BLP, respectively), but we can equally well analyse RTs obtained in studies on other languages. The only restriction for our approach is that the RT distributions per word-frequency band should be constructed with high fidelity, which is increasingly possible with the rise of megastudies.

In conclusion, SDA is a powerful and versatile methodology that fits experimental RT distributions to simple probability distributions as a function of item properties. It enables researchers to find answers to key theoretical questions like those concerning the impact of word frequency and word length on RTs and the quantitative split of RTs between the deterministic nondecision time and the stochastic integration of information. More concretely, SDA revealed better performance of (a) contextual diversity measures over word form frequency and (b) the lnorm distribution over the Wald and gamma distributions to describe RT distributions. The superiority of contextual diversity measures raises an important point. In the past, items were often considered in isolation and characterised by a potentially small set of properties. However, it has become increasingly clear that word processing relies on many properties, including those that extend beyond the actual word as exemplified by contextual diversity. SDA and its Bayesian implementation is well suited to incorporate a large numbers of variables in the analysis of RTs, including continuous variables such as AoA and neighbourhood densities. As shown in the methods section, and in the code provided with this manuscript, SDA is straightforward to apply even for large datasets. This makes it ideal to study big data comprised of millions of RTs and a large set of explanatory variables. SDA is also ideal for comparing probability distributions across conditions and populations, which is timely as we move towards embracing the heterogeneity and diversity of study participants. Quantifying individual differences will be key to building predictive and generalizable models that will be invaluable to researchers in their quest to identify and elucidate the mechanisms that underlie visual word recognition.
